# Improving the knowledge of labour and delivery nurses in India: a randomized controlled trial of mentoring and case sheets in primary care centres

**DOI:** 10.1186/s12913-016-1933-1

**Published:** 2017-01-07

**Authors:** Janet Bradley, Krishnamurthy Jayanna, Souradet Shaw, Troy Cunningham, Elizabeth Fischer, Prem Mony, B. M. Ramesh, Stephen Moses, Lisa Avery, Maryanne Crockett, James F. Blanchard

**Affiliations:** 1 Centre for Global Public Health, Faculty of Medicine, University of Manitoba, 771 Mc Dermot Avenue, Medical Rehabilitation Building, Room R070, Winnipeg, MB R3E 0T6 Canada; 2 Karnataka Health Promotion Trust, IT Park 5th floor, #1-4 Rajajinagar Industrial Area, Behind KSSIDC Admin Office, Rajajinagar, Bangalore, 560 044 India; 3 IntraHealth, 6340 Quadrangle Drive, Suite 200, Chapel Hill, NC 27517 USA; 4 St. John’s National Academy of Health Sciences, Sarjapur Road, Bangalore, Karnataka State 560 034 India

**Keywords:** Maternal health, Neonatal health, Mentoring, Supervision, India, Birth checklists, Patient case sheets

## Abstract

**Background:**

Birthing in health facilities in India has increased over the last few years, yet maternal and neonatal mortality rates remain high. Clinical mentoring with case sheets or checklists for nurses is viewed as essential for on-going knowledge transfer, particularly where basic training is inadequate. This paper summarizes a study of the effect of such a programme on staff knowledge and skills in a randomized trial of 295 nurses working in 108 Primary Health Centres (PHCs) in Karnataka, India.

**Methods:**

Stratifying by district, half of the PHCs were randomly assigned to be intervention sites and provided with regular mentoring visits where case sheet/checklists were a central job and teaching aid, and half to be control sites, where no support was provided except provision of case sheets. Nurses’ knowledge and skills around normal labour, labour complications and neonate issues were tested before the intervention began and again one year later. Univariate and multivariate analyses were conducted to examine the effect of mentoring and case sheets.

**Results:**

Overall, on none of the 3 measures, did case sheet use without mentoring add anything to the basic nursing training when controlling for other factors. Only individuals who used both case-sheets and received mentoring scored significantly higher on the normal labour and neonate indices, scoring almost twice as high as those who only used case-sheets. This group was also associated with significantly higher scores on the complications of labour index, with their scores 2.3 times higher on average than the case sheet only control group. Individuals from facilities with 21 or more deliveries in a month tended to fare worse on all 3 indices. There were no differences in outcomes according to district or years of experience.

**Conclusions:**

This study demonstrates that provision of case sheets or checklists alone is insufficient to improve knowledge and practices. However, on-site mentoring in combination with case sheets can have a demonstrable effect on improving nurse knowledge and skills around essential obstetric and neonatal care in remote rural areas of India. We recommend scaling up of this mentoring model in order to improve staff knowledge and skills and reduce maternal and neonatal mortality in India.

**Trial registration:**

This study is registered at clinicaltrials.gov, Identifier No. NCT02004912, November 27, 2013.

**Electronic supplementary material:**

The online version of this article (doi:10.1186/s12913-016-1933-1) contains supplementary material, which is available to authorized users.

## Background

Maternal mortality in India remains among the highest in the world, despite a reduction from 570 in 1990 to less than 200 per 100,000 in 2013 [1]. The main causes of maternal mortality are postpartum haemorrhage, sepsis, obstructed labour, abortion and hypertensive disorders [2], with the first four accounting for 60% of maternal deaths [3]. It has been observed that a key barrier to safe delivery is timely and appropriate emergency obstetric care once women have reached a facility in labour, sometimes referred to as the “third delay”, and that 86% of the cause of the this delay is inadequate staff training, drug procurement issues, lack of equipment, staff shortages and low staff motivation [4]. Basic obstetric skills at the first level of care are essential for preventing maternal deaths [5].

India also has a high neonatal mortality rate, at 35 per 1000 live births (42.5 in rural areas) [6]. In 2005, there were 2.3 million child deaths in India, 20% of all child deaths in the world [7], and 900,000 neonatal deaths [8]. One-third of neonatal deaths occur on the first day [9, 10], when infants are often still in health institutions. Seventy-eight percent of neonatal deaths are attributed to three main causes: prematurity and low birth weight; neonatal infections; and birth asphyxia [6, 8].

Birthing in health facilities has increased over the last few years (there was a 57% rise in institutional deliveries between 2005 and 2008) since the creation of the National Rural Health Mission (NRHM) in 2005 (now called the NHM – National Health Mission) and its Janani Suraksha Yojana (JSY) cash incentives scheme. Nevertheless, maternal and neonatal mortality rates remain high [11]; there was only a 2.5% drop in perinatal maternal mortality in the 2005–2011 period leading to the suggestion that the quality of care in those facilities is poor [11, 12]. George, in a study in Karnataka state also concluded that women seek care but are often inappropriately diagnosed and/or managed [12]. A cornerstone of the NHM effort to improve labour and delivery has been the appointment of new staff nurses to conduct services at “24x7” primary health centres (PHCs), providing them with three-week intensive skilled birth attendant (SBA) training, in addition to basic training [13]. However, there is no specific midwife cadre; this means that nurses have been described as “circumstance driven midwives” where they have to do midwifery among all other jobs, have little specific training, little authority, power or responsibility, lack professional satisfaction and basically cope with the situation [14]. There is also poor follow-up or support after this training and little documentation of how nurses recognize and manage obstetric and neonate problems [15]. In the developed world, clinical mentoring is viewed as essential for the training of health workers [16, 17], and in other contexts studies have pointed to the need for high quality supportive supervision and mentoring of staff after training, particularly where basic training may be inadequate [18, 19]. A survey of nurses in northern Karnataka, India, showed a poor grasp of basic labour and delivery issues and concluded that supportive supervision and enhanced clinical mentoring should be implemented, as well as user-friendly case sheets or checklists that could be used as job-aids and also for documentation [20]. This paper summarizes a study of the effect of case sheets with and without mentoring on nurse knowledge and skills in a randomized trial of 108 PHCs over a one year period in Karnataka, India.

## Methods

Two districts in northern Karnataka state, south India, with 108 PHCs, were selected for the study. All sites were provided with a supply of newly developed comprehensive patient case sheets introduced at a three-day orientation session for staff nurses before the start of the main mentoring intervention. The case sheet was designed for use with women in labour from arrival to discharge. It includes sections on initial assessment, labour monitoring, delivery and postpartum care. It also has a set of sheets for identification and pre-referral management of complications that detail appropriate drugs and essential actions. Then stratifying by district, half of the PHCs were randomly assigned to be intervention sites and provided with regular mentoring visits (with case sheets integrated as a job and teaching aid) and half to be control sites, where no support was provided. By providing case sheets and/or on-site mentoring, it was hypothesized that the knowledge and skills of providers to conduct normal deliveries and to identify and manage complications would improve, over and above their basic government skilled birth attendant (SBA) training.

The mentoring intervention addressed improvements in both clinical practice and service delivery through a dedicated cadre of nurse mentors (NMs). Eleven nurse mentors, each responsible for 5–6 intervention PHCs, were recruited and trained for 5 weeks in essential clinical competencies and in how to effectively mentor PHC staff in clinical knowledge and skills, team building, problem solving and service delivery improvement. The NMs visited each of their assigned intervention PHCs for 2–3 days every two months starting in August 2012 for a total of six times during the first year. They used tools and approaches such as self-assessments, observations, clinical and case sheet audits, and interviews as aids to make an assessment of capacities in the facilities. They attempted to upgrade staff nurse knowledge and skills through case reviews, demonstrations and modeling of good practice, bed-side case discussions, and small group teachings on such issues as partograph use, management of normal labour and complications management. Apart from clinical mentoring, the mentors focused on team building and self-assessment problem-solving around all aspects of the provision of quality maternal, neonatal and child health (MNCH) services.

Data collection involved interviews with all available staff nurses (SNs) in all 108 facilities. A questionnaire was designed and field tested twice for a total of 8 days before being finalized (Additional file 1). Closed ended questions on key aspects of labour, delivery and the postpartum period, focused on identification and pre-referral management of critical complications: some aspects of knowledge were tested using case studies (such as asking staff to read and interpret 23 questions about a filled partograph) and some questions required them to demonstrate a skill, such as neonatal resuscitation with an ambu bag and mask. Simultaneous data collection was carried out in both study districts over a period of one month in April 2012 (before the 3 day case sheet orientation) and again in August 2013 (12 months after the actual start of the main mentoring intervention). Data were entered and analyzed using IBM SPSS version 22 (IBM Corporation, Armonk, New York, 2013).

### Sample size calculation

Assuming that 10% of staff nurses had correct knowledge (of all three steps of active management of third stage of labour) at the time of baseline and that the intervention would improve it to at least 20%, setting alpha level error at 0.05 (95% confidence limits), beta level error set at 0.2 (80% power) and intra class correlation at 0.4, the study required an estimated sample of 54 clusters and 162 staff nurses per arm. The two study districts had 109 functional 24/7 PHCs at the time of study. One facility was under renovation at the time of the study, and so 108 were included in the study, of which 54 were randomly allocated into an intervention arm and 54 into a control arm. Within the facilities, all nursing staff available were interviewed in the study and were the unit of evaluation.

### Statistical analyses

#### Main analyses

First we conducted basic univariate analyses comparing staff and site characteristics at both time periods, examining explanatory variables associated with exposure to interventions (use of case sheets with and without mentoring), and other potential confounding factors (client caseload, nursing experience and district).

#### Sub analyses

In order to more fully understand improvements in the follow-up time period, we selected several variables for grouping into three major domains: Normal Labour, Complicated Labour and Neonatal Issues, based on their association with maternal and neonatal mortality in India. Composite indices for the three knowledge domains were created by entering relevant test items into a principal components analysis (PCA); due to the binary nature of all test items, polychoric PCA was used. PCA is a variable reduction technique that reduces a set of observed variables into a smaller set of uncorrelated components (i.e. *principal components*) which account for most of the variance in the observed variables. Thus, each component is a linear combination of the observed variables optimally weighted to account for the maximum amount of variance. For each knowledge domain, all components with an eigenvalue of greater than 1 (>1) were kept as outcome variables for further analyses.

Following PCA, and for each index developed, linear regression using generalized estimating equations (GEE) was used to assess the association between type of support received and performance on the index. Thus, separate multivariable regression models were estimated for each of the knowledge domains. GEE was used to correct for clustering by the facility from which each individual was recruited. Available confounders added to multivariable models included average number of deliveries in the facility per month (21 or more vs. fewer than 21); the districts (Bellary vs. Gulbarga); and the number of years since basic nurse training (1–5 years vs. 6 or more years). Stata V12 (College Station, TX) was used for PCA and regression analyses.

Ethical approval for the study was obtained from the Institutional Ethical Review Board of the St John’s Medical College and Hospital, Bangalore, India. Written informed consent was obtained from all participants before interview.

## Results

### Main analyses

#### Respondent characteristics and response rates

In the 2012 baseline survey, before the start of the mentoring program, we interviewed 295 SNs, 90% of whom were recently employed NHM contractual staff nurses (SNs). Nurses were young, with 69% qualified for less than 6 years (mean 5.2 years). In the 2013 survey we interviewed 273 SNs; 47% had been qualified for less than 6 years (mean 6.1 years); 92% had received the government 21 day Skilled Birth Attendant (SBA) training, up from 80% in the baseline survey.

#### Provider knowledge

Several aspects of provider knowledge essential for managing women and babies during the perinatal period were examined. In 2012, despite four-fifths of nurses having had SBA training, knowledge was very poor, with little difference between intervention and control sites (Table 1), and no difference between the 80% of staff who had attended SBA training and the 20% who had not, scoring only better on 2 of the 13 indicators examined (data not shown). Few could correctly identify common complications such as prematurity, obstructed labour, eclampsia or foetal distress; few knew how to manage the third stage of labour to prevent haemorrhage, perform neonatal resuscitation, use a partograph, or manage obstructed labour or low birth weight babies. The majority could name the correct drugs for active management of the third stage of labour (AMTSL), hypertension and eclampsia, but knowledge of appropriate drugs for maternal puerperal sepsis and neonatal infections was very low.

**Table 1:** Staff knowledge of essential aspects of maternal and neonatal care, comparing those from intervention and control sites, Karnataka, India, 2012 and 2013

	Baseline, 2012 (*n* = 295)		Post intervention, 2013 (*n* = 273)	
	Control (%) (*n* = 154)	Intervention (%) (*n* = 141)	Control (%) (*n* = 137)	Intervention (%) (*n* = 136)
Can define prematurity correctly	4.3	4.3	21.9ǂ	46.3ǂ*
3 aspects of AMTSL correct	9.1	6.4	35.8ǂ	82.4ǂ*
Partograph score >16/23 questions correct	0	0	34.3ǂ	74.3ǂ*
AMTSL drug correct	69.5	68.8	87.6ǂ	95.6ǂ*
3 signs of eclampsia correct	1.9	1.4	16.8ǂ	47.8ǂ*
3 main signs of obstructed labour correct	0	0	8.8ǂ	28.7ǂ*
All 3 maternal sepsis drugs correct	0.6	2.1	10.9ǂ	73.5ǂ*
Eclampsia drug correct	75.3	77.3	86.1ǂ	94.9ǂ*
Hypertension drugs correct	57.1	55.3	76.6ǂ	83.1ǂ
FHR distress upper and lower ranges correct	16.2	11.3	37.2ǂ	65.4ǂ*
3 main aspects of LBW management correct	9.1	14.9	40.9ǂ	58.1ǂ*
All 4 aspects of neonatal resuscitation correct	2.6	2.1	11.7ǂ	48.5ǂ*
2 neonate sepsis drugs correct	4.5	2.9	3.6	44.9ǂ*

ǂ Difference between control sites in 2012 and 2013 and between intervention sites in 2012 and 2103 (*p* < 0.05)

*Difference between intervention and control sites in 2012 and between intervention and control sites 2013 (*p* < 0.05)

One year later, staff knowledge had increased significantly in both types of sites. However, the effect was significantly more noticeable in the mentoring intervention sites, where often 2 to 3 times more staff than in control sites could give the correct answers. Importantly, more staff were able to recognize danger signs, but were also much more aware of the appropriate drugs to use and of how to manage AMTSL, obstructed labour, neonatal resuscitation and low birth weight. Many gaps remained, however, even in intervention sites, especially with respect to defining prematurity, recognizing obstructed labour, eclampsia and foetal distress, managing neonatal sepsis and obstructed labour, and resuscitating babies with asphyxia.

We were interested in what other factors might affect knowledge improvement in 2013, and also to try to explain why control site staff knowledge had also increased over time, albeit not to the same extent. We examined years since basic training as we had noted during the programme that older nurses seemed to have difficulty learning the new SBA standards. We also examined the exposure nurses had had to training and other project interventions: nurses had received SBA training; nurse worked in a facility that reported use of case sheets (whether intervention sites or not); nurse worked in a facility that had received the mentoring intervention. We also looked at site delivery caseload, as anecdotal observations in the field had suggested that staff working in the larger PHCs were struggling to cope with a large number of deliveries and had little time to learn new things.

There were very few differences between earlier and more recently qualified nurses, although the recently qualified did slightly better on most indicators (Table 2). Unlike in 2012, there were significant differences in 2013 between those nurses who had received SBA training and those who had not; scoring statistically significantly better on 7 of the 13 parameters. Our hypothesis that staff in smaller sites were more knowledgeable proved to be correct; they scored generally better than staff in busier PHCs. There were significant differences between staff in sites that noted using case sheets and those that did not, but of course, some of these nurses also received mentoring and some did not.

**Table 2:** Knowledge of various aspects of maternal and newborn care by potential explanatory factors, all sites, 2013 (*n* = 273)

Variables	21 day SBA training (%)		Years since basic nursing training		Case sheets used in PHC (%)		Mentoring in PHC (%)		Monthly delivery caseload at PHC	
Categories	No (23)	Yes (250)	0–5 years (172)	6+ years (101)	No (78)	Yes (194)	No (137)	Yes (136)	0–20 (195)	More than 20 (78)
Can define prematurity correctly	30.4	34.3	33.7	34.7	25.3	37.6*	21.9	46.3*	34.4	33.3
3 aspects of AMTSL correct	39.1	60.8*	60.5	56.4	35.4	68.6*	35.8	82.4*	58.5	60.3
Partograph score >16/23 questions correct	30.4	56.4*	58.7	46.5*	34.2	62.4*	34.3	74.3*	57.9	44.9*
AMTSL drug correct	73.9	93.2*	90.7	93.1	88.6	92.8	87.6	95.6*	91.8	91.0
3 main signs of obstructed labour correct	4.3	20.0	20.9	14.9	6.3	23.7*	8.8	28.7*	17.4	21.8
3 signs of eclampsia correct	13.0	34.0*	32.6	31.7	13.9	39.7*	16.8	47.8*	34.4	26.9
All 3 maternal sepsis drugs correct	43.5	42.0	45.9	35.6	17.7	52.1*	10.9	73.5*	44.1	37.2
Eclampsia drug correct	69.6	92.4*	89.0	93.1	83.5	93.3*	86.1	94.9*	91.8	87.2
Hypertension drugs correct	60.9	81.6*	77.3	84.2	78.5	80.4	76.6	83.1	82.1	74.4
FHR distress upper and lower ranges correct	26.1	53.6*	54.1	46.5	35.4	57.7*	37.2	65.4*	55.4	41.0*
3 main aspects of LBW management correct	30.4	51.2	53.5	42.6	35.4	55.2*	40.9	58.1*	47.7	53.8
All 4 aspects of neonatal resuscitation correct	13.0	31.6	29.7	30.7	16.5	35.6*	11.7	48.5*	30.3	29.5
2 neonate sepsis drugs correct	17.4	24.8	24.4	23.8	5.1	32.0*	3.6	44.9*	27.2	16.7

*p < 0.05

### Sub-analyses

#### Principal components and multivariable regression analyses

Composite indices for three knowledge domains that reflect elements of essential maternal and newborn care, were created using principal components analysis (PCA). The first principal component was used for each of the three knowledge domains, as all of the first components had eigenvalues greater than 1 and accounted for the majority of the variance in each of the knowledge domains. The proportion of variance explained by the first principal component was 0.57, 0.60 and 0.54 for the Normal Labour, Complications of Labour, and Neonatal Issues domains, respectively (not shown). Table 3 shows the characteristics of the 250 nurses who had received SBA training that were included in the multivariable model. Table 4 shows the results from the regression model with knowledge of normal labour, labour complications and neonatal complications indices used as the outcome variables. All analyses were adjusted for level of intervention (basic SBA training only, training plus case sheets and training plus case sheets and mentoring), number of deliveries in a month, district and years since nurse training.

**Table 3:** Descriptive characteristics of explanatory variables, 2013 (*n* = 250)^a^

	No.	%
Nurse exposure to interventions		
SBA training only	67	26.8
SBA training, case sheets, no mentoring	62	24.8
SBA training, case sheets and mentoring	121	48.4
Monthly delivery in facility		
0–20	179	72.0
21+ deliveries per month	71	28.0
Years since basic nursing training		
0–5 years	157	62.8
6+ years	93	37.2
District		
Bellary	122	48.8
Gulbarga	128	51.2

^a^The 23 nurses who had not received SBA training were not included in this analysis

**Table 4:** Association between interventions and other variables, and three knowledge domains based on multivariable analysis (*n* = 250): (*p*-values)

		Normal labour index^a^	Labour complications index^b^	Neonatal complications index^c^
Interventions	SBA training only	*Ref*	*Ref*	*Ref*
	SBA training, case sheets, no mentoring	0.043 (0.77)	0.044 (0.76)	0.119 (0.46)
	SBA training, case sheets and mentoring	1.055 (<0.01)**	1.370 (0.00)**	1.227 (<0.01)**
Monthly deliveries at facility	0–20 per month	*Ref*	*Ref*	*Ref*
	21+ per month	−0.226 (0.06)	0.383(0.00)**	−0.270 (0.04)*
Years since basic nurse training	6+ years	*Ref*	*Ref*	*Ref*
	0–5 years	−0.019 (0.85)	0.110 (0.25)	0.015 (0.88)
District	Bellary	*Ref*	*Ref*	*Ref*
	Gulbarga	−0.058 (0.59)	0.021 (0.84)	0.172 (0.14)

*p < 0.05; **p < 0.01. Based on a linear regression using GEE analysis

^a^Normal labour index included knowledge of the following items: Prematurity; all 3 aspects of active management of the third stage of labour; partograph score; and oxytocin use

^b^Labour complications index included knowledge of the following items: Eclampsia signs; obstructed labour signs; maternal sepsis drugs; eclampsia drugs; and hypertension drugs

^c^Neonatal complications index included knowledge of the following items: Upper and lower bounds for foetal heart rate; low birth weight management; neonatal resuscitation; and newborn sepsis drugs

Overall, on none of the 3 measures, did case sheet use without mentoring, add anything to SBA training alone when controlling for other factors. Only individuals who used both case-sheets and received mentoring scored significantly higher on the normal labour index (*p* < .001), scoring almost twice as high as those who only used case sheets. Similarly, only those from the group who had case sheets and mentoring, scored significantly higher on the neonatal issues index (*p* < .001), scoring almost twice as high as the those who used case sheets but had no mentoring. Lastly, this group was also associated with significantly higher scores on the complications of labour index (*p* < .001), with their scores 2.3 times higher on average than the case sheet group. Individuals from facilities with 21 or more deliveries in a month tended to fare worse on all 3 indices, particularly complications of labour index (*p* < .0001) and neonatal complications (*p* < 0.05). There were no differences in outcomes according to district or years of experience.

## Discussion

Supervision plays a key role in performance but is frequently characterized by periodic inspection and control rather than support and feedback to improve performance [21]. However, for the last few years, there has at least been an understanding that *supportive* supervision should emphasize joint problem solving, mentoring and two way communication, address weaknesses in the supporting health systems, provide professional development, job satisfaction and motivation, model good practice, and give information about guidelines and standards of practice. In turn, supervisors need to be well trained, equipped and have transport [17, 19]. Implementing such an intensive quality improvement programme is not easy, especially in a large country like India with thousands of health facilities, a largely rural population, isolated health workers and poor infrastructure. There have been few randomized trials of such initiatives [19]. Our study of 108 facilities and almost 300 nurses in two large rural districts showed that in the space of one year and only 6 visits, the supervision/mentoring project had a demonstrable effect on knowledge and skills. We attribute this to a well-trained cadre of sensitive, non-threatening nurse mentors, good central support and funds for travel, a philosophy of teamwork, self-assessment and problem solving, and good quality on the job training and mentoring, with use of the case sheet as a central hook on which to hang teachings and case reviews [22]. The study confirmed that staff in busier PHCs were in fact not as knowledgeable as staff in smaller centres, and this allowed us to change the mentoring strategy and focus more attention there in year two of the programme.

In many countries it has been assumed that poor health worker performance reflects inadequate knowledge and skills. As a result most interventions have focused on training, with mixed and sometimes disappointing long term-results [19]. Training is clearly important, but most now acknowledge that there is a need to move beyond training with multi-faceted interventions [19]. Certainly our 2012 baseline data showed that SBA training without additional inputs appeared to have had a limited effect on nurses’ knowledge and skills, although the additional programmatic inputs in 2013 did seem to have strengthened knowledge of those who had the SBA training over those who had not.

In a study reviewing 15 studies of interventions to improve health worker performance, it was concluded that the dissemination of written guidelines (or case sheets) alone without added interventions is also ineffective [19]. Our study showed clearly that although provision of case sheets and a 3 day orientation, added to the knowledge of nurses in univariate analysis, it did not make them perform significantly better than those who had only had SBA training, when controlling for other factors in the multivariable analysis. This was not unexpected as the case sheets are of necessity long and detailed. Without proper training they might be somewhat difficult to follow, and without adequate encouragement, might appear cumbersome. Furthermore, the case sheets offer guidance/protocols in how to manage complications and much of this guidance actually differs from long standing practices. In the mentoring sites, the case sheets were used as job aids, but also as teaching tools during every mentoring visit, so that their use over time was understood to be relevant and important for proper patient management. Some have suggested that short reminder-based checklists might be useful, and one study found they could be successfully implemented in a sub-district hospital in India [23]. We would argue that without good basic knowledge, these checklists still might not be understood or provide sufficient guidance, and without mentoring, their use would not be reinforced.

We do not know for sure why knowledge and skills improved among staff in the control sites in 2013, albeit not to the same extent as staff from the intervention sites. However, it is possible that a combination of factors might explain this effect: more staff receiving basic SBA training; improved government SBA training; attendance at a 3 day case sheet orientation; some case sheet use; cross-pollination of ideas between staff in different PHCs; or encouragement by district officials who discussed the mentoring and case sheet in district meetings attended by staff from all PHCs.

There is evidence that women in India are now opting for delivery in facilities; the proportion of institutional deliveries increased from 42% in 2004 to 69% in 2009 [24]. However, Rao [25] and Prasad [26] note that nursing education in India has suffered neglect. A survey showed 61% of nursing colleges were unsuitable for teaching, with an acute shortage of faculty and facilities [27]: inadequate libraries and demonstration rooms, overworked teaching staff, little practical experience for students and few opportunities for in-service training for teaching staff [28]. Training institutions in many states have even shut down even as NHM seeks to induct more nurse midwives [29]. While improvements to pre-service training are needed in the longer term, innovations to enhance the knowledge and practices of those nurses already in situ are urgently needed.

The study had some limitations. First it should be noted that outcomes are measured at the individual knowledge level within sites; thus, the overall performance of intervention versus control sites, with respect to reductions in maternal and neonatal mortality, was not assessed. Second, we did not assess actual nurse practices as this was beyond our scope and budget. Certainly knowledge is a precursor to good practice and without it we might assume practice is thus poor. However more research is needed to know what should be the key areas for focus in training and mentoring programs and their measurement.

## Conclusions

This study demonstrates that provision of case sheets alone is insufficient to improve knowledge and practices. However, on-site mentoring in combination with case sheets can have a demonstrable effect on improving nurse knowledge and skills around essential obstetric and neonatal care in remote rural areas of India. We recommend scaling up of this mentoring model in order to reduce maternal and neonatal mortality in India.

## Additional file

Additional file 1: Participant questionnaire. Questionnaire administered to all staff nurses working in all 108 study facilities. (DOC 268 kb)

## Abbreviations

AMTSL: Active management of the third stage of labour
GEE: Generalized estimating equations
JSY: Janani Suraksha Yojana
MNCH: Maternal, neonatal and child health
NHM: National Health Mission
NM: Nurse mentors
NRHM: National Rural Health Mission
PCA: Principal components analysis
PHC: Primary health centres
SBA: Skilled birth attendant
SN: Staff nurse
