# Supplementary material for: Improving the knowledge of labour and delivery nurses in India: a randomized controlled trial of mentoring and case sheets in primary care centres
Source: BMC Health Serv Res. 2017 Jan 7;17:14. doi: 10.1186/s12913-016-1933-1 (PMC5219705; doi:10.1186/s12913-016-1933-1)
Supplement: Additional file 1: — Participant questionnaire. Questionnaire administered to all staff nurses working in all 108 study facilities. (DOC 268 kb) [file 12913_2016_1933_MOESM1_ESM.doc]

**Sukshema Project**

**MNCH Mentoring evaluation**

Interview with staff nurses in 24/7 PHCs

| **Response** | | | | **Code** |
| --- | --- | --- | --- | --- |
| **DISTRICT:**   1. BELLARY 2. GULBARGA | | | |  |
| **TALUKA NAME:**............................................................... | | | |  |
| **PHC NAME:**...................................................................... | | | |  |
| **PROVIDER**   1. STAFF NURSE (REGULAR) 2. STAFF NURSE (CONTRACTUAL) | | | |  |
| **SEX OF PROVIDER**   1. FEMALE 2. MALE | | | |  |
| **INTERVENTION SITE? (FILLED BY SUPERVISOR)**   1. Yes 0. No | | | |  |
| **RESULT OF INTERVIEW:**   1. COMPLETED 2. POSTPONED 3. NOT COMPLETED 4. REFUSED 5. OTHER (SPECIFY)................................................ | | | |  |
| **DATE OF ASSESSMENT:** DAY MONTH YEAR | | | | |
| **NAME AND SIGNATURE OF INVESTIGATOR**................................................................... | | | |  |
| **INFORMED CONSENT OBTAINED**   1. YES 0. No | | | |  |
| **TYPE OF ASSESSMENT**   1. Pre-intervention 2. Post intervention 3. Routine monitoring | | | |  |
|  | **Spot Checked** | **Office Edited** | **Data Entry** | |
| **Name:** |  |  |  | |
| **Date:** |  |  |  | |
| **Signature:** |  |  |  | |

I would just like you to tell me a few things about yourself and your training and then I will ask you a few questions about how you do your job. Don’t worry about any of this – only I will know your answers. The results are going to be used to improve training and support.

| **Q No** | **Question** | | | | **Response** | | **Skip to** | |  | |
| --- | --- | --- | --- | --- | --- | --- | --- | --- | --- | --- |
| A | How many years ago did you finish your basic GNM/BSc training? | | | |  | |  | |  | |
| B | Have you had SBA 21 day training from the government? | | | | Yes..............................1  No...............................0 | | Q.D | |  | |
| C | If so, how many YEARS ago? | | | |  | |  | |  | |
| D | Did you attend any **govt** SBA 3-4 day refresher training | | | | Yes..............................1  No...............................0 | | Q.F | |  | |
| E | If yes, how many MONTHS ago | | | |  | |  | |  | |
| F | For how many years have you worked in this facility | | | |  | |  | |  | |
| G | Total number of years you have been working as a Staff Nurse | | | |  | |  | |  | |
| H | **ONLY FOR 2013 survey**  Did you attend the SBA refresher training organized by Sukshema/SJMC in 2012? | | | | Yes..............................1  No...............................0 | |  | |  | |
|  | |  | |  | |  | |  | | |
| **Section 1 - Management of labour and complications** | | | | | | | | | | |
| Q.No | **Question** | | **Coding categories** | | | | | | | **Skip to** |
| 1 | Can you tell me how many weeks gestation is a full term pregnancy? | | Note number of weeks | | | | | | |  |
| 2 | Women in pre-term labour need referral – at less than how many weeks gestation? | | Less than how many weeks | | | | | | |  |
| 3 | Can you tell me what is the 3^rd^ stage of labour?  Is it.......?  *Read the list and write the code in box* | | 1. Early latent phase of labour 2. Active phase of labour 3. Delivery of the baby 4. Delivery of the placenta 5. The immediate postpartum period   *Comments* | | | | | | |  |
| 4 | Can you tell me what are the 3 key components of the “Active Management of the Third Stage of Labour” (AMTSL) in a singleton birth?  *Do not read, but ask, “anything else”?*  *Code Yes/No* | | Yes No   1. Give a uterotonic /oxytocin/pitocin/syntocin 1 0 2. Do controlled cord traction OR (cord traction   and counter traction) for delivery of placenta 1 0   1. Do fundal massage 1 0 2. Other............................................................ 1 0   *Comments* | | | | | | |  |
| 5 | It is important to give a uterotonic drug to prevent haemorrhage. When should you give this?  *Do not read. Code Yes or No* | | Yes No   1. Immediately after anterior shoulder delivery 1 0 2. Immediately after baby is out 1 0 3. Other............................................................ 1 0   *Comments* | | | | | | |  |
| 6 | What would be the drug of choice to ***give before the placenta is out*** to prevent haemorrhage?  *Do not read.*  *Write the Code 1 or 0 in box* | | 1. Mentions oxytocin/pitocin/syntocin? 2. Other (Specify).................................................   *If oxytocin etc. not mentioned, skip the next question*  *Comments* | | | | | | |  |
| 7 | If the response is (1), ask what dose and what route.  *Do not read answers. Note down the answers given and code yes/no* | | CORRECT  Yes No   1. Dose………...10 IU (or 2 ampoules or 2 mls) 1 0 2. Route................................... IM 1 0   *Comments* | | | | | | |  |
| 8 | When a woman is in labour at your PHC, with what maternal/fetal conditions should you refer her to a higher facility?  Do not read the list but ask, “anything else?”  Code Yes/No | | Yes No   1. Eclampsia 1 0 2. Pre-eclampsia 1 0 3. Significant bleeding (Antepartum or   postpartum haemorrhage) 1 0   1. Gestation less than 37 weeks 1 0 2. Severe sepsis 1 0 3. Prolonged or obstructed labour 1 0 4. Multiple pregnancy 1 0 5. Previous C-section 1 0 6. Severe anaemia 1 0 7. Mal-presentation 1 0 8. Premature rupture of membranes   and no labour after 12 hours 1 0   1. Severe illness such as diabetes,   heart disease, asthma 1 0   1. Fetal distress 1 0 2. Other......................................................... 1 0   *Comments* | | | | | | |  |

| 9 | Here is a partograph of a woman in labour (Case study attached).  Rani (wife of Ramu), 18 years of age, was admitted at 10:00 am on 11 Jan 2012 with complaints of 39 weeks pregnancy and labour pains since 7:00 am. This is her first pregnancy.  Please refer to the partograph and answer the following questions: | **(WRITE IN ANSWERS GIVEN – CODE IF CORRECT)**  Yes No   1. What was the foetal heart rate at admission?   140 per minute ………………………… 1 0   1. What was the dilatation of cervix on admission?   4cms …….……… 1 0   1. What were her vital signs on admission?   (all 3 have to be correct to score 1)  BP……………Pulse………………….Temp…………….  BP 100/70 Pulse 80 per min Temp 37.8. 1 0   1. When did the membranes rupture?   12 noon ……………………………………………….. 1 0   1. Describe the contractions at 12 noon.   ………………………………………………………..  3 per 10 minutes 1 0  Medium strength 1 0   1. What was the foetal heart rate at 2 pm?   160 per min ……………………………. 1 0   1. What was the cervical dilatation at 2 pm?   6cms ……………………………………….. 1 0   1. What was the colour of amniotic fluid at 2 pm?   Meconium stained …………………………………… 1 0   1. List what things you need to check at 2pm   ..................................................................................   1. Dilatation 1 0 2. Foetal heart 1 0 3. Amniotic fluid 1 0 4. Mother’s vital signs 1 0 5. Contractions 1 0   **10.** What action will you take in Rani’s case at 2pm?  ……………………………………………………………………………………………………………………..…………………………………………………………………………..  Yes No   1. Give IV fluids                                                  1    0 2. Give ampicillin                                               1 0 3. Give metronidazole                                       1      0 4. Give gentamicin     1 0 5. Give oxygen 1 0 6. Refer   1 0 7. Why will you take this action? What is the problem?   ……………………………………………………………………………………………………………………………………………………………………………………………………………………………………………………………..  Yes No   1. Prolonged (or obstructed labour or   failure to progress) labour 1 0   1. Fetal distress 1 0 2. Probability of infection 1 0   **Total Score Q.9 (out of 23)............................**  *Comments* |  |
| --- | --- | --- | --- |
| 10 | How do you know if a woman has eclampsia? What are the three signs?  *Do not read the list but ask, “anything else?”*  *Code Yes/No* | MENTIONED  Yes No   1. High BP ( >140/90) 1 0 2. Proteinuria 1 0 3. Convulsions or loss of consciousness 1 0 4. Other..................................... 1 0   *Comments* |  |
| 11 | If you have a woman in labour with a BP of 160/110, protein in urine and having convulsions, what is the **ONE key drug of choice** should you give her to control her convulsions?  *Do not read the list. Ask for only ONE answer and write the code in box.* | 1. Magnesium sulphate 2. Diazepam 3. IV fluids 4. Ampicillin 5. Oxytocin 6. Sodium bicarbonate 7. Nothing 8. DK 9. Other....................................................   *If magnesium sulphate not mentioned, skip the next question*  *Comments* |  |
| 12 | If the response is magnesium sulphate, ask what dose and what route  *Do not read answers. Note down the answers given and code yes/no* | CORRECT  Yes No  1. Dose.....................20mls or 10mg or 10 amp. 1 0  2.Route........................................IM or IV 1 0  *Comments* |  |
| 13 | What else should you do or give before you refer her? Tell me everything you should do or give to her if she has high BP and convulsions.  *Do not read the list but ask, “anything else?”*  *If they say anti- hypertensive, ask what drug? Circle if hydralazine and/or nifedipine mentioned*  *Code Yes/No* | Yes No  a. Hydralazine or nifedipine (or Depin) 1 0  b. clear her airway 1 0  c. start oxygen 1 0  d. start IV line 1 0  e. place Foley catheter 1 0  f. Other................................................. 1 0  *Comments*  I*f hydralazine or nifedipine not mentioned, skip the next question. Circle if hydralazine or nifedipine and then ask about ONLY the drug mentioned in the next question* |  |

| 14 | If the response includes either hydralazine or nifedipine, ask what dose and what route  *Only ask about the drug they have mentioned above*  *Do not read answers. Note down the answers given and yes/no* | CORRECT  Yes No   1. Hydralazine 2. Dose......................................5mg 1 0 3. Route................................... IV 1 0 4. Nifedipine (or Depin) 5. Dose......................................5mg 1 0 6. Route................................... oral 1 0   *Comments* |  |
| --- | --- | --- | --- |
| 15 | Jyoti is a 30 year old gravida 5 para 4. She just gave birth at your PHC to a healthy full term baby weighing 2.6kg. You practiced AMSTL and gave oxytocin as the baby was delivered. The placenta was delivered intact 5 minutes later, but after 30 minutes, Jyoti is still bleeding heavily.  What will you check immediately?  *Do not read the list but ask, “anything else?”*  *Code Yes/No* | Yes No   1. Vital signs – pulse, BP 1 0 2. Check for uterine tone/contracted uterus 1 0 3. Estimate the blood loss 1 0 4. Check for trauma/tears 1 0 5. Other.............................................................. 1 0   *Comments* |  |
| 16 | You find that Jyoti is pale. Her pulse is 108 per minute, BP is 80/60mmHg, and the bleeding is heavy (1 pad soaked in 5 minutes). Her uterus is soft and relaxed. There are no vaginal tears.  What are **all** the things you need to do or give before you refer her?  *Do not read the list but ask, “anything else?”*  *Code Yes/No* | Yes No   1. Put in IV line with Ringer lactate 1 0 2. Give oxytocin 1 0 3. Massage the uterus 1 0 4. Start BIMANUAL compression of uterus 1 0 5. Raise her legs higher than her head 1 0 6. Keep her warm with a blanket 1 0 7. Monitor pulse and BP every 15 minutes 1 0 8. Encourage her to pass urine /catheterize 1 0 9. Other........................................................... 1 0   *Comments* |  |
| 17 | How do you know that a woman after delivery has a vaginal or uterine infection? What are the 4 main signs?  *Do not read the list but ask, “anything else?”*  *Code Yes/No* | Yes No   1. Fever >38^0^C 1 0 2. Lower abdominal pain or tenderness 1 0 3. Abnormal lochia (foul-smelling or   blood-stained) 1 0   1. Uterus not well contracted 1 0 2. Other................................................ 1 0   *Comments* |  |
| 18 | If you suspect a serious uterine infection, what drugs do you need to give her, before you refer her?  *Do not read the list but ask, “anything else?”*  *Code Yes/No* | Yes No   1. Ampicillin (or ampicillin+ cloxacillin   or ampilox or ampoxin) 1 0   1. Metronidazole 1 0 2. Gentamicin 1 0 3. Other..................................................... 1 0   *Comments*  *Only ask the next question if they mention any of these 3 drugs. If they mention NONE of these, skip the next question* |  |
| 19 | If the provider mentions 1, 2 or 3, ask what dose and what route?  *Only ask about the drugs they mentioned in the previous question*  *Do not read answers. Note down the answers given and code yes/no* | CORRECT  Yes No Not  asked   1. Ampicillin (or amp+clox or ampilox or ampoxin) 2. Dose....................................................1g 1 0 9 3. Route.............................IV or oral 1 0 9 4. Metronidazole 5. Dose ...............................400mg or 500mg 1 0 6. Route...........................................oral or IV 1 0 7. Gentamicin 8. Dose...........................................80mg (2mls) 1 0 9. Route.................................................IM or IV 1 0   *Comments* |  |
| 20 | How do you know that a woman is in obstructed labour? What are the 5 main signs?  *Do not read the list but ask, “anything else?”*  *Code Yes/No* | Yes No   1. Strong contractions, no progress in   dilatation, no descent of presenting part 1 0   1. Partograph is below/ right/cross the alert line 1 0 2. Fetal distress 1 0 3. Rapid maternal pulse 1 0 4. Horizontal ridge across uterus below umbilicus 1 0 5. Other......................................................... 1 0   *Comments* |  |
| 21 | If a labour is obstructed, what should you do or give before you refer her?  *Do not read the list but ask, “anything else?”*  *Code Yes/No* | Yes No  1. Give IV fluids 1 0  2. Give Ampicillin (or ampicillin+ cloxacillin  or ampilox or ampoxin) 1 0  3. Give Metronidazole 1 0  4. Give Gentamicin 1 0  5.Give oxytocin (wrong answer) 1 0  6. Other.......................................................... 1 0  *Comments*  *Only ask the next question about if they mention ampicillin (and alternatives) or metronidazole or gentamicin. If they mention NONE of these, skip the next question* |  |
| 22 | If the provider mentions 2 or 3, or 4 ask what dose and what route?  *Only ask about the drugs they mentioned in the previous question*  *Do not read answers. Note down the answers given and code yes/no* | CORRECT  Yes No Not  asked  1. Ampicillin (or amp+clox, or ampilox or ampoxin)  a. Dose....................................................1g 1 0 9  b. Route.............................IV or oral 1 0 9  2. Metronidazole  a. Dose ...............................400mg or 500mg 1 0  b. Route...........................................oral or IV 1 0  3. Gentamicin  a. Dose...........................................80mg (2mls) 1 0  b. Route.................................................IM or IV 1 0  *Comments* |  |
| 23 | If a foetus is in distress what is its heart rate (upper and lower)?  *Note down the answers given and code yes/no* | Yes No   1. Foetal heart lower rate <120 ....................... 1 0 2. Foetal heart upper rate >160........................ 1 0 3. Other .......................................................... 1 0   *Comments* |  |
| 24 | What is the definition of a low birth weight baby? Under how many grams?  *Write Code 1 or 0 in box* | 1. Less than 2500 gms 2. Other.....................................................   *Comments* |  |
| 25 | What should you do if the baby is <1800 gms at birth? Tell me all the things you would do.  Do not read the list but ask, “anything else?”  Code Yes/No | Yes No   1. Keep baby warm 1 0 2. Initiate kangaroo/skin to skin care   as soon as possible 1 0   1. Refer to facility that can deal with LBW babies 1 0 2. Other......................................................... 1 0   *Comments* |  |
| 26 | What are the most important things to monitor in the mother in first 2 hours after delivery?  *Do not read the list but ask, “anything else?”*  *Code Yes/No* | Yes No   1. Pulse 1 0 2. BP 1 0 3. Uterine fundus tone/uterus contracted 1 0 4. Vaginal bleeding 1 0 5. Other......................................................... 1 0   *Comments* |  |
| 27 | What are some of the danger signs that you should be looking for in a woman **between birth and the first 2 days?** Tell me anything about a woman that would make you think she was unwell  *Do not read the list but ask, “anything else?”*  *Code Yes/No* | Yes No   1. Fever >38c 1 0 2. Breast , nipple, feeding problems 1 0 3. Pain in abdomen 1 0 4. Foul discharge 1 0 5. Excessive bleeding 1 0 6. Headache 1 0 7. Convulsions/ confusion /consciousness   blurred vision 1 0   1. Burning urination or blood in urine 1 0 2. Swollen part of leg 1 0 3. Pain in perineum 1 0 4. Others......................................................... 1 0   *Comments* |  |

| **Section 2 – Managing newborns** | | | |
| --- | --- | --- | --- |
| 28 | If this baby is not breathing and needs resuscitation, what will you do? Please show me using this doll, what would you do, and describe what you are doing as you do it, tell me for how long you do it, step by step.  *Do not read the list*  *Code Yes/No if the provider does any of these (or says they are doing them)* | CORRECT  Yes No   1. Positions baby with neck extended 1 0 2. Positions mask properly, holding with   thumb and one finger, and 2 fingers  on baby’s chin and ensures good seal 1 0   1. Squeezes the bag 40-60 breaths   per minute or by saying “squeeze,  two, three, squeeze” 1 0   1. Assesses chest expansion after   30 seconds 1 0  *Comments* |  |
| 29 | What are some of the danger signs that you should be looking for in a newborn baby **between birth and the first 2 days?** Tell me anything about a baby that would make you think it was unwell.  *Do not read the list but ask, “anything else?”*  *Code Yes/No* | Yes No   1. Breast feeding difficulty 1 0 2. No urination in first 48 hours 1 0 3. No meconium in 24 hours 1 0 4. Cyanosis 1 0 5. Irritable or lethargic 1 0 6. Hypothermia 1 0 7. Hyperthermia 1 0 8. Jaundice-palm and sole 1 0 9. Breathing fast/difficult 1 0 10. Convulsions/ irregular or   jerky movements 1 0   1. Abdominal distension 1 0 2. Cord stump red or discharging 1 0 3. Persistent diarrhea / vomiting 1 0 4. Bleeding from any site 1 0 5. Other....................................................... 1 0   *Comments* |  |

| 30 | If you thought a baby had a severe infection in the first 2 days what would you do or give?  *Do not read the list but ask, “anything else?”*  *Code Yes/No* | Yes No   1. Give ampicillin 1 0 2. Give gentamicin 1 0 3. Refer 1 0 4. Other................................................. ...... 1 0   *Comments*  *Only ask the next question if they say ampicillin or gentamicin. In not, skip next question* |  |
| --- | --- | --- | --- |
| 31 | If provider answers 1 or 2, then ask what dose and what route?  *Only ask about the drugs mentioned in the previous question*  *Do not read answers. Note down the answers given and code yes/no* | CORRECT  Yes No   1. Ampicillin 2. Dose........................50mg/Kg 1 0 3. Route.........................IM 1 0 4. Gentamicin 5. Dose...........................5mg/Kg 1 0 6. Route.................................IM 1 0   *Comments* |  |

| **Section 3 – Preparing for going home** | | | | |
| --- | --- | --- | --- | --- |
| 32 | When the mother takes the baby home, what should you tell her about cord care?  *Do not read the list*  *Code Yes/No* | Yes No   1. Put nothing on it 1 0 2. Apply chlorohexidine 1 0 3. Apply turmeric 1 0 4. Apply coconut oil 1 0 5. Other ............................................1 0   *Comments* |  | |
| 33 | What other information do you give women before they are discharged?  *Do not read the list*  *Code Yes/No* | Yes No   1. Breast feeding advice 1 0 2. Danger signs in mother 1 0 3. Danger signs in baby 1 0 4. Come back if problem 1 0 5. Take iron and folic acid 1 0 6. Keep baby warm by swaddling 1 0 7. Cover baby’s head 1 0 8. Give no bottles, cow’s milk,   water or tea 1 0   1. When to bath the baby 1 0 2. About immunizations 1 0 3. About family planning 1 0 4. When to return for check up 1 0 5. Other.................................................. 1 0   *Comments* |  | |
| 34 | What immunizations and medicines do you **ALWAYS** give a newborn **BEFORE** discharge from the PHC after delivery?  *Do not read the list*  *Code Yes/No* | Yes No   1. OPV 1 0 2. Hep B 1 0 3. BCG 1 0 4. Vitamin K 1 0 5. Others...................................... 1 0   *Comments* |  | |
| **Section 4 – Skills building and attitudes** | | | | |
| 35 | Has anyone helped you in the last year with hands-on, on the job, practical skills building in the following areas?  **Read the list**  Code Yes/No for each one | MENTIONED  Yes No   1. How to use a partograph? 1 0 2. How to do AMTSL? 1 0 3. How to manage complications   of labour? 1 0   1. How to resuscitate a baby? 1 0 2. How to manage complications in   newborns? 1 0   1. How to keep good records? 1 0 2. How to work to good quality   standards? 1 0   1. Others................................................. 1 0   *Comments* | |  |
| 36 | Out of 100% how would you rate the people who come from outside (supervisors etc) in helping you improve clinical care for mothers and babies? | **SCORE....................................**  *Comments* | |  |
| 37 | Out of 100% how would you score the following?  Read the list | **SCORE**   1. I feel part of a highly functional team......................... 2. I feel confident in delivering babies   here at the PHC ...........................   1. My opinion is truly valued by other   staff at the PHC ...........................   1. I really enjoy my job at the PHC ............................   *Comments* | |  |

38. Is there anything you would like to tell me about labour and delivery and care of newborns at this PHC? ***(Please write below, full sentences in English).***

***________________________________________________________________________________________________________________________________________________________________________________________________________________________________________________________________________________________________________________________________________________________________________________________________________________________________________________________________________________________________________________________________________________________________________________________________________________________________________________________________________________________________________________***

**39.** Interviewer please write down any comments /information of interest gleaned during the interview. Write in clear English.

**Case study -THE SIMPLIFIED PARTOGRAPH AT PHC LEVEL (N.B use new partograph from patient case sheet in 2013)**

**IDENTIFICATION DATA**

**Name:** Rani

**Date & Time of Admission**

11th Jan 2012, 10 AM

**W/e:** Rambhanaj

**Date & Time of ROM:**

11th Jan 2012, 12 Noon

**Age:** 18 yrs **Parity:** Primigravida **Reg. No.:**

Foetal heart rate

200

|  |  |  |  |  |  |  |  |  |  |  |  |  |  |  |  |  |  |  |  |  |  |  |  |
| --- | --- | --- | --- | --- | --- | --- | --- | --- | --- | --- | --- | --- | --- | --- | --- | --- | --- | --- | --- | --- | --- | --- | --- |
|  |  |  |  |  |  |  |  |  |  |  |  |  |  |  |  |  |  |  |  |  |  |  |  |
|  |  |  |  |  |  |  |  |  |  |  |  |  |  |  |  |  |  |  |  |  |  |  |  |
|  |  |  |  |  |  |  |  |  |  |  |  |  |  |  |  |  |  |  |  |  |  |  |  |
|  |  |  |  |  |  |  |  |  |  |  |  |  |  |  |  |  |  |  |  |  |  |  |  |
|  |  |  |  |  |  |  |  |  |  |  |  |  |  |  |  |  |  |  |  |  |  |  |  |
|  |  |  |  |  |  |  |  |  |  |  |  |  |  |  |  |  |  |  |  |  |  |  |  |
|  |  |  |  |  |  |  |  |  |  |  |  |  |  |  |  |  |  |  |  |  |  |  |  |
|  |  |  |  |  |  |  |  |  |  |  |  |  |  |  |  |  |  |  |  |  |  |  |  |
|  |  |  |  |  |  |  |  |  |  |  |  |  |  |  |  |  |  |  |  |  |  |  |  |
|  |  |  |  |  |  |  |  |  |  |  |  |  |  |  |  |  |  |  |  |  |  |  |  |
|  |  |  |  |  |  |  |  |  |  |  |  |  |  |  |  |  |  |  |  |  |  |  |  |
|  |  |  |  |  |  |  |  |  |  |  |  |  |  |  |  |  |  |  |  |  |  |  |  |

190

180

170

160

150

140

130

120

110

100

90

80

**(A) Foetal Condition**

| I | I | I | I | C | C | M | M | M |  |  |  |  |  |  |  |  |  |  |  |  |  |  |  |
| --- | --- | --- | --- | --- | --- | --- | --- | --- | --- | --- | --- | --- | --- | --- | --- | --- | --- | --- | --- | --- | --- | --- | --- |

Amniotic fluid

10

9

8

Cervix (cm) 7

[Plot X] 6

5

4

**(B) Labour**

Hours

1 2 3 4 5 6 7 8 9 10 11 12

Time

10 AM

11 AM 12 Noon 1 PM 2 PM

| Contractions      per 10 min | 5  4  3  2  1 |
| --- | --- |

**(C) Interventions**

Drugs and

[V] fluids given

**(D) Maternal Condition**

Pulse and BP

180

170

160

150

140

130

120

110

100

90

80

70

60

Temp

| 37.8 |  |  |  |  |  |  | 37.6 |  |  |  |  |  |  |  |  |  |  |  |  |  |  |  |
| --- | --- | --- | --- | --- | --- | --- | --- | --- | --- | --- | --- | --- | --- | --- | --- | --- | --- | --- | --- | --- | --- | --- |
